# Transactions of the Medical and Physical Society of Bombay for the Years 1842 and 1843

**Published:** 1846-04

**Authors:** 


### Art. IV.

*Transactions of the Medical and Physical Society of Bombay for the Years* 1842 and 1843. pp. 196; pp. 250.—*Bombay,* 1842-3.

In the present days of bookmaking, when the press is teeming with works so numerous that it would require a person's undivided time to peruse them, periodical literature assumes an importance which under other circumstances could not be claimed for it. It becomes the channel through which many, whose avocations necessarily occupy a large portion of their time, become acquainted with what is going on in the literary and scientific world, and who, by the notice taken of the emanations from the press, are led either to read them as being valuable contributions to the information on the subjects of which they treat, or to pass them over as undeserving of the time required for a careful perusal. On this account 'Reviews' in the present day deservedly occupy a very prominent position. But there is another branch of periodical literature which also is of much value, comprising those 'Journals' set apart for original communications, and the 'Transactions' of learned societies. By means of these, observations, cases, and papers, which although valuable in themselves, and often leading to important results, would not be of sufficient consequence to warrant the publication of a monograph, are brought under the notice of the profession. This class of periodicals is particularly available to officers in the public service, who enjoy many excellent opportunities of collecting facts and making important observations, but who from the numerous demands upon their time, especially in unhealthy colonies, are unable to work up their materials into a volume, and even did circumstances permit this, are unwilling to risk the expense of publication, especially when at a distance, and unable to superintend it themselves.

The volumes now before us we consider to be of high value, as affording a channel through which medical officers in India can make known to the profession the results of their observations in that wide and rich field for the cultivation of physical and medical science, which has hitherto been much neglected or at least but partially improved. Considering the disadvantages under which these officers labour, from the numerous demands upon their time, the amount of mental exertion required in the discharge of their duties, and the enervating effects of the climate, we cannot but look upon these Transactions as highly creditable to all concerned in their production.

The Medical and Physical Society of Bombay was established in November 1835, its principal object being to collect and disseminate information on the medical and physical sciences. In furtherance of these views, it was proposed to publish, half-yearly if practicable, a volume containing, "1st, original communications, whether received from members, or from government, or from the medical board, which shall be recommended for publication by the committee of papers; 2d, a report for the past half-year, drawn up by the secretary of the society from such documents as may have been placed at the disposal of the society by government or the medical board,—this report to be read at a meeting of the society before publication; and 3d, an appendix consisting of selections of interest from medical works and periodicals." The society seems to have received every assistance from the authorities; the local government having assented to the transmission of all letters on the business of the society, under the signature or to the address of its secretary, free of postage; and having given permission to have its circulars printed at the government press. The medical board and deputy-inspector-general of hospitals also became patrons of the society, and placed at its disposal the returns and reports in their offices, while the same facilities were afforded them by the medical authorities at Madras. Under such auspices the society could scarcely fail of success. Some modifications have been found necessary in its arrangements, but on the whole it seems to be in a flourishing condition. An annual volume has been published instead of a half-yearly, and the proposed report by the secretary does not appear to have been drawn up. We do not doubt that the society has been productive of much good by stimulating the industry of the medical officers in the Presidency. It is a matter of regret that a similar volume of transactions has never been got up in this country by the army medical department, which might easily be done were similar encouragements held out by the government and medical board as in India.

We do not propose to enter into a detailed consideration of the papers contained in the volumes before us, but shall briefly notice one or two of the most important. Before doing so we may remark, that in the fifth volume is given a recapitulation of the titles of the papers in the four preceding, a reference to which will show those interested in the subject the nature of the contributions which this society has been the means of eliciting.

The first paper we shall notice is a "Medico-historical abstract of the first year's service in the East Indies of H. M. 14th regiment of Light Dragoons at Kirkee, under the Bombay Presidency, by J. W. Moffat, esq. surgeon, H. M. 14th Light Dragoons." Kirkee is the only station in this Presidency provided with permanent accommodation for a regiment of European cavalry. It is about 60 miles up the country E.S.E. from Bombay, at an elevation of 1820 feet above the level of the sea, and is celebrated as the spot where, in 1817, Mr. Elphinstone's subsidiary force scarcely numbering 3000 Sepoys, under Colonel Burr, repulsed a sudden attack made upon them by upwards of 20,000 Mahrattas. The barracks were erected in 1827, are well constructed, and calculated to afford excellent accommodation for a regiment 700 strong. There is a good hospital, built in 1830, capable of holding 120 patients, and allowing 1100 cubic feet of space to each.* Besides a good description of the station, this paper contains many judicious remarks on the causes of diseases to which soldiers are more particularly exposed on their first arrival in India; the best means of avoiding or diminishing these, and the plan of treatment which our author found most successful. He has drawn up some excellent tables, showing the strength and deaths of the different ranks, and the influence of age and length of service in India upon the mortality. We do not intend to enter upon these points as the data are insufficient, and the period of observation too short, to warrant positive deductions being drawn, but if similar abstracts are furnished by the medical officers at Kirkee for a series of years, they will afford the means of elucidating several still controverted points. We trust that by thus calling attention to them, officers at other stations may be induced to follow Mr. Moffat's example.

In treating of the interior economy of the regiment our author says:

"Tables and forms are provided also for the convenience of messing, and a scale of diet is laid down, and furnished by government at a reduced rate to the soldier. The daily ration drawn for each man, not in hospital nor in imprisonment, is—bread, 1 lb., second sort; beef, 1 lb.; rice, 4 oz.; salt, 2 oz.; coffee or tea, $\frac{5}{7}$ oz.; sugar, $1\frac{1}{2}$ oz.; firewood, 3 lbs.; with one dram of arrack at the option of the soldier, to be consumed at three regular meals, breakfast, dinner, and supper. The men are messed in squads of four, and native cooks are entertained at a small weekly cost, who are further engaged to provide milk for the tea or coffee, butter, eggs, steaks, chops, additional vegetables, cheese, &c., as they may be occasionally asked for by varying tastes and appetites. The station being in such proximity to a populous city, (Poona, $2\frac{1}{2}$ miles distant,) all sorts of supplies are procurable in abundance, and little could be altered in the ration for the advantage of the men beyond the occasional variety of mutton. The supply of vegetables is probably wisely left to the choice of the messes; and for the rice, which is not much relished, over and above what is used for the soup, unless when curried meat is the order of the day, the cooks are not averse to substitute carrots, onions, country greens, and occasionally pumpkins, yams, &c. A dram of spirits still continues to be offered to the men at dinner time, and an inducement to take it is held out in its issue at a lower rate by one fourth than its ordinary retail price. The issue of a ration dram is a relic of former times, when salt meat and stale provisions were the ordinary and only rations provided; but surely it may be well dispensed with when mixed provisions of an excellent and wholesome quality are supplied, and its continuance leads to a wrong impression of its utility and necessity."

The spirit ration has been long abolished in our other possessions, and we can see no good reason for its being retained in India. It is true the soldier is not now as formerly compelled to swallow it or to throw it away, lest he should sell it to some thirsty comrade, but as he is offered it at a lower rate than he could purchase it at, he looks upon it as part of the reward for his services, and rather than give up any of his rights he

* The author of the paper states 1100 cubic *inches*, but this is evidently a mistake. He has fallen into the same error regarding the space in barracks, which he states at 1400 cubic *inches*. By a calculation made from the dimensions of the rooms, as given in this paper, the cubic space to each man appears to be about 1000 feet.

accepts it. By this practice not only is the intemperate confirmed and encouraged in his vicious habits, but the young soldier is induced to commence a system of dram-drinking, acquires a fondness for it, and is soon converted into a drunkard, unfit to be trusted, a disgrace to the service, a burden to himself, and a nuisance to every one connected with him. We have frequently heard officers inveigh against the debauchery and drunkenness of the soldier in India, but is not much of the blame attributable to those who thus tempt him onward in his career of intemperance? To Sir Henry Hardinge the army is deeply indebted for the abolition of the spirit ration in 1830, and most sincerely do we trust that he will take advantage of his present exalted position to extend this boon to the troops in India, and to remove finally and completely this source of disease, insubordination, and crime.

A ration of spirits was originally issued from a mistaken idea that it conduced to the preservation of health, but we fear it is not issued now from any idea of benefit to the soldier, but for the sake of the large profits accruing from the canteens, and the duty on arrack, to the Hon. Company of Merchants trading to the East Indies. If our conjecture is right we may well say, in the words of Lord Liverpool, "This is too bad."

The above description offers a very enviable picture of the condition of the cavalry soldier in India, at least when not on field service. To illustrate the great improvement that has taken place in this respect, through the force of public opinion, and the gradual progress of civilization, we subjoin a statement, by one who had practical experience of it, of the mode of messing troops in Ceylon in 1803, and we have reason to believe the description was applicable, at that time, to India. Of the truth of this account we entertain no doubt, although, contrasting it with the preceding, it seems scarcely credible, because we know that the same system was in operation fifteen years afterwards: it has only been during the last twenty-five years that these improvements have been effected.

"When the meat was brought to the cooking-place, it was thrown down upon a dirty mat, and chopped up, then the cooks sat down upon their hams, placing a knife between their toes, and cut it up into small pieces; and thus daubed all about, and without even being washed, it was boiled in curry, the rice being boiled at the same time in another earthen vessel, called a chattie, as all their boiling is done in earthen vessels, and then brought into the barracks at twelve o'clock, when the soldiers gathered round them like as many voracious hounds, with their chatties in their hands, bawling blasphemy, and grumbling, not so much at the quality as the quantity of their food.

"At night we got what was called supper, which consisted of a small cake, made of rice flour and water, and a liquid called coffee, although there was not a single grain of the berry in it. What we used for sugar was called jaggery (a coarse kind of sugar, made in Ceylon, from the cocoa-nut,) which was made up in cakes, very insipid and dirty; it bore no resemblance to the sugar used in Europe. At eight o'clock in the morning we got breakfast brought to us in the same manner; it consisted of the same cake, fish, or bullock's liver, and jaggery-water, and this formed the daily diet of the British troops in Ceylon. Had it been of good quality, and properly cooked, it was well enough: our allowance of liquor, which was arrack, was one quart per day, to five men, or about two drams to each.

"The beef, of which we had a pound per day, was given out along with the rice in the morning, for which sixpence per day was kept off our pay. But it was rather carrion than beef. The cattle, sometimes buffaloes, sometimes bullocks, having been used in their husbandry, were generally lean, old, or diseased; the meat was soft, flabby, and full of membraneous skin; it had a rank, heavy, loathsome odour, was offensive to both sight and smell, and hurt me more than the climate, and was, I am certain, the cause of much of the disease and death which thinned our numbers. The rice, too, was small and of a bad quality, full of dust and dirt, from which our rascally cooks were at no trouble to free it."*

Shades of the 19th regiment, think of butter, eggs, steaks, chops, cheese, carrots, onions, pumpkins, and yams!!!

The principal mortality in the 14th Light Dragoons, during its first year in India, arose from epidemic cholera, by which 22 were cut off. But for this unfortunate circumstance the deaths would have been very little above the proportion incident to a regiment at home, amounting only to 14 from all causes, out of a strength of 650. The diseases most prevalent were fevers, chiefly of the continued type, and the treatment appears to have been very successful, only 1 case in 76 of this class having proved fatal. The most important feature in them was a marked predisposition to the symptoms of *delirium tremens*, which perhaps in no small degree arose from the habitual use of ardent spirits, sanctioned and encouraged by authority, as already noticed.

The diseases next in frequency, were diarrhœa and dysentery, which, however, were not of a very fatal character, owing probably to the sound constitution of the men being as yet unimpaired by the climate; we fear that subsequent years will show a much less favorable result. We would strongly recommend a perusal of this paper to young medical officers going to India, both as containing much information and many useful hints, and also as an example worthy of being followed. In concluding our notice of it, we deeply regret to add that its author has since fallen a victim to the Indian climate.

Dr. A. S. Thomson has entered at considerable length into the very interesting and important question, "Could the natives of a temperate climate colonize and increase in a tropical country, and vice versa." To elucidate this he has collected from various sources as much information as he could upon the results of the successive attempts at colonization in India, by the Portuguese, Dutch, English, French, and Danes, and by the Dutch and Spaniards in the Indian Archipelago. He also reviews the state of the European colonies in tropical Africa and tropical America, and has brought together a large amount of statistical evidence. From these data, which would occupy too much space to lay before our readers, he concludes that "there is little doubt the tropical parts of the world are not suited by nature for the settlement of natives of the temperate zone. European life in these parts is with difficulty prolonged, much sickness is suffered, and their offspring become degenerate and cease to propagate their species in a few generations; and should necessity force Europeans to perform the drudgery of labouring in the field, their lives will be rendered still shorter, and their existence will be little better than a prolonged sickness."

The evidence on the practicability of natives of a tropical country colonizing a temperate climate is more limited, but would appear to

* The Life of Alex. Alexander, p. 109.

justify our arriving at the same conclusion. In addition to the fact of the great mortality in the 4th West India regiment, when removed to Gibraltar in 1819, we find that in Philadelphia the deaths among the blacks amount to nearly 5 per cent., or 1 in every 21 living, and in New York to 1 in every 22 living, a rate of mortality which must eventually terminate in the extinction of the black population, unless it is kept up by immigration. From a careful consideration of the evidence he has collected, Dr. Thomson infers,

"That man, both from his mental and physical structure, is able to resist sudden vicissitudes of climate better and for a greater length of time than any of the inferior animals, yet he is only born to flourish in climates analogous to that under which his race exists, and that any great change is injurious to the increase and to the mental and physical development of man. The nearer the climate of the original resembles the adopted country, so much the less will be the injurious effect . . . . . There is a theory in medicine denominated the doctrine of *acclimatization* or *seasoning*, the principles of which are, that the human constitution, after a certain length of time, becomes suited to any great change of food, climate, or indeed any unusual mode of life. On this theory is founded the opinion that the first few years of an European's residence in the tropics are more fatal than any other period, an opinion opposed to many statistical facts and at variance with the statements adduced in this paper; for it must be obvious if the doctrine of *seasoning* were correct, the European ought to be completely fitted in the course of sixty years to resist every injurious effect of the tropics, and that their offspring should almost resemble the aboriginal races in being able to bear the heat, a result perfectly opposed to almost every fact which has been quoted.

With the exception of a few barren islands, the whole surface of the globe at present known is peopled by the human race. This diffusion of man from the poles to the equator must have been extremely gradual, an opinion which an examination of the sacred and profane history of the world proves. . . . . . No great vicissitude of climate or sudden change of food or mode of living was therefore experienced, and the human race increased and multiplied on the earth."

In the fifth volume Dr. Thomson has given a "history of the epidemic fever which prevailed among the men of H. M. 17th regiment during the monsoon of 1841, when quartered in the Colabah barracks, Bombay," which we notice because it affords a striking example of the benefit derived from sending the sick to sea. 166 sick, all of whom had suffered from fever, were embarked on the 27th of October, and cruised northward to the Persian gulf, and arrived at Bombay on the 24th of November. "The sick, embarked emaciated and without appetite, soon began to eat and get strong; almost every man had a paroxysm or two of fever during the cruise, but mild in its nature compared to what they had at Colabah." No case proved fatal at sea, and very few of them were severe.

We are surprised that an officer who understands the principles of statistics, as Dr. Thomson has proved he does, should have omitted entirely to state the strength of the regiment during the prevalence of this epidemic, as it must be obvious that a mere statement of the number of cases admitted into hospital conveys very indefinite information when the amount of the force among whom they occurred is not given at the same time.

In the fifth number of the Transactions is an article, entitled, "Reports on the medical statistics of Upper Scinde, drawn up by the medical officers serving with the force under the command of Brigadier England. Presented by the medical board, November 1842." These reports were called forth by the following

"MEMORANDUM FOR MEDICAL OFFICERS. The medical officers of this force are to be called on by the assistant adjutant-general to report, for the information of the supreme government of India, on all points regarding the medical statistics of their respective stations. 1. The climate generally as regards the troops. 2. The average number of soldiers per cent. under medical treatment, during the worst months and during the best. 3. The seasons, showing the best and worst for the troops, and whether the past season has been in any way a peculiar one. 4. The number of deaths during the last four months, out of men. 5. The sort of accommodation which the troops have had, and specifying how far sickness has been influenced thereby. 6. The particular months during which each station is most unhealthy, and the causes. 7. The unwholesomeness, generally of particular places. 8. The medical officers are to be directed to make these documents as complete as possible, and to offer such suggestions, as to clothing, &c., with a view to remedy the evils discovered, as may be most likely to lead to the attainment of that desirable object. By order, (signed) W. WYLLIE, Major A.A.A. General, Scinde Force. Camp, Quetta, 6th September, 1841."

We believe it is not customary to criticise the literary composition of orders issued by the military authorities, especially when on service; but we may remark that the gallant major might, without much trouble, have written (if, indeed, he wrote it at all,) a more grammatical and more intelligible memorandum. But we have still more important faults to find: it was not issued till the beginning of September, and we have no reason to suppose the medical officers had any previous intimation that they would be called upon to afford information on these points. During the period respecting which they were to report, therefore, many opportunities of investigating these subjects may have been allowed to pass unheeded, and when at length the Memorandum was issued, the officers may have been obliged, in the absence of recorded observations, to trust to their memory and the impressions left by events long past. Surely such a document should have been issued at the commencement of the expedition.

We are not informed whether this memorandum was issued by the advice of the medical board, or whether any medical officer was consulted as to the information most likely to be useful, or the form in which it ought to be called for; but it is very evident that whoever drew it up must have been extremely ignorant of the principles of statistical investigations. For instance, the officers are ordered to report "the average number of soldiers per cent. under treatment," but this information is of very little value unless we know the amount of the force; one or two unsound men in a very small body of troops might by frequent readmissions into hospital increase the ratio per cent. to such an extent as to lead to the inference (and it might be a very erroneous one) that the post was unheathy, while such a circumstance would scarcely affect the results if the numbers were considerable. Again, they were to report "the number of deaths during the last four months out of men," (!!!) a statement which would not convey the slightest information of practical utility as the strength was not given. A simple monthly numerical return of the strength, the admissions into hospital, and deaths, with the diseases by which these were occasioned, would have been much more useful than the reports furnished, as from it the authorities could have obtained all the information called for in Nos. 2, 3, 4, 6, and 7, while a few remarks appended to it on the climate, barrack and hospital accommodation, diet, and clothing, would have rendered it tolerably complete, with the additional advantage of the results being in a form which would admit of their being compared with each other. It may be remarked, that of the nine reports in this volume, one only, that of surgeon Patch, 21st N. I. and superintending surgeon S. P. contains the information in the form we have above suggested.

We may be supposed to have dwelt too much on the imperfections of these reports, but we have done so not from useless regret for a lost opportunity of collecting valuable information on the influence of a climate hitherto unknown, but in the hope that similar opportunities may not be thrown away through mis-directed exertions. Scarcely a year now passes that some expedition is not fitted out in India to be sent into parts of that extensive empire hitherto unexplored or but little known, and it is much to be regretted that due advantage is not taken to procure, in a systematic form, the observations of the medical officers accompanying the troops,—a class of men who from their education and general acquirements are well qualified to carry out scientific investigations. We have been informed that it is intended to adopt in the Indian army, official medical returns similar to those which have been so long in use in the British army; this will go far to remedy existing defects, by introducing a uniform system throughout the service, and will render the information obtained from every different quarter available for the purposes of comparison. But we trust the East India Company will not rest satisfied with merely organizing a set of returns, but will make the necessary arrangements for having them properly condensed and published annually, with a selection from the observations in the accompanying reports. If they are to be consigned to the dusty shelves of a government office for want of duly qualified persons being appointed to reduce them to a practical shape, the medical officers will soon discover this, and make them out in any way that causes least trouble; indeed, under such circumstances they might just as usefully send in a quire or two of blank paper. Among the objects sought for by the establishment of statistical returns in the army may be enumerated two: 1st, that of affording to the military authorities information regarding the efficiency of the troops at the various stations, the circumstances affecting their efficiency, and the best means of promoting and increasing it; and 2d, of furnishing to the medical officers the results of the experience of their professional brethren as to the salubrity of different situations, the causes of disease, and the hygienic and therapeutic measures best adapted for the maintenance of the soldiers in an efficient condition. To effect the last of these objects a volume should be published annually, giving in a condensed form the information contained in the returns and reports, a copy of which should be transmitted to each medical officer in the army. There can be no doubt that this would prove a powerful stimulus to officers to exert themselves to collect information of a useful and interesting nature, as it would be the means of bringing their names favorably under the notice of the profession.

It is much to be regretted that no arrangements have ever been made to render available the vast mass of valuable reports which have been accumulating for the last thirty years at the medical board in this country. A portion of the numerical returns has been condensed and published in the "Blue Books," on the Health of the Army, but the medical histories of diseases, &c. still lie there, nor, so far as we can learn, is there any prospect of their being made any use of. We trust such will not be the case in India, but that coeval with the adoption of a system of returns and reports will be the establishment of a department to render them available to the military authorities and to the medical profession.

In the 6th No. is a series of "Cases illustrative of the pathology of the diseases of Bombay, by Dr. C. Morehead," the secretary of the society, which appear to have been drawn up with much care. The paper was commenced in the 2d No. of the Transactions, in which 45 cases were detailed; in the present number he has added 68 to these; but as he states that he proposes in a future volume to make the practical remarks on pathology and treatment which a consideration of them, in connexion with cases of similar diseases successfully treated, is calculated to suggest, we shall reserve our observations till the series is completed.

Having thus noticed a few of the more important papers in these Transactions, we may in conclusion express our hearty wishes for the society's prosperity. It has in the volumes before us given practical proof of its utility as a medium of eliciting and communicating valuable information, and we trust that as years roll on, it will continue to collect and disseminate still more valuable fruits than it has hitherto done. We had almost forgotten to add, that while the matter is good, the style of getting up these volumes is very creditable to all concerned.

